# Baseline Clinician Attitudes and Practices Regarding Long-Term Neurological Follow-Up for COVID-19 Patients: Early Pandemic Insights With Contemporary Relevance

**DOI:** 10.7759/cureus.103837

**Published:** 2026-02-18

**Authors:** Kathryn N Becker, Mackenzie Hagood, Justin F Creeden, Holly Heck, Peterson T Haak, Cristin Larder, Claire Tipton, Jason Schroeder, Kathryn M Eisenmann

**Affiliations:** 1 Neurosurgery, University of California San Diego, San Diego, USA; 2 Neurosurgery, The University of Toledo College of Medicine and Life Sciences, Toledo, USA; 3 Psychiatry, University of California San Diego, San Diego, USA; 4 Medical Education, The University of Toledo College of Medicine and Life Sciences, Toledo, USA; 5 Epidemiology and Biostatistics, Michigan State University College of Human Medicine, East Lansing, USA; 6 Epidemiology and Biostatistics, Larder Data Consulting, LLC, East Lansing, USA; 7 Medical Education/Cell and Cancer Biology, The University of Toledo College of Medicine and Life Sciences, Toledo, USA

**Keywords:** covid-19, covid-19 and neurology, long covid, neurological effects of coronavirus, pandemic response, survey research

## Abstract

Objectives:* *This study aimed to investigate clinician opinions and practices regarding long-term neurological follow-up for COVID-19 patients during the early pandemic period (June-August 2020), before formal long COVID recognition, and identify the gaps between clinician awareness and clinical practice implementation.

Methods:* *We conducted a cross-sectional survey of 199 physicians and advanced practice providers (APPs; including nurse practitioners, physician assistants, certified registered nurse anesthetists (CRNAs), and similar roles) across Northwest Ohio and Southeast Michigan healthcare systems (June-August 2020). The survey assessed clinician awareness of long-term neurological risks, screening behaviors, referral practices, and attitudes toward long-term neurological follow-up using an original adaptive questionnaire administered via Qualtrics. Bivariate associations were tested using Pearson’s chi-square and Fisher’s exact test (p ≤ 0.05). This study was approved by the University of Toledo Institutional Review Board (Protocol #300681, exempt status).

Results:* *Most respondents (54/96, 56.3%) believed that more than 50% of COVID-19 patients should receive long-term neurological follow-up, primarily by neurology specialists (66/134, 49.3%). However, only 13/93 (14.0%) were actively referring more than 50% of their COVID-19 patients for such care. Average referral rates increased modestly from 14.1% pre-pandemic to 19.9% during the pandemic, with 100/130 (76.9%) reporting no change in practices. Self-reported familiarity with neurological manifestations of COVID-19 (114/179, 63.7%) was substantially lower than general COVID-19 familiarity (178/180, 98.9%).

Conclusions:* *Substantial disconnect existed between clinician beliefs about appropriate care and actual referral practices, revealing infrastructure barriers, including inadequate specialty capacity and lack of standardized protocols. These baseline findings document the state of clinical practice before formal long COVID guidelines were established.

Policy implications:* *Healthcare systems require enhanced infrastructure for long-term neurological monitoring of COVID-19 survivors, including increased specialty capacity and standardized protocols, informing pandemic preparedness for chronic disease complications.

## Introduction

Since its emergence in late 2019, COVID-19 has resulted in over 777 million confirmed cases and more than 7 million deaths worldwide, with the true toll estimated to be substantially higher [1]. While the acute crisis has subsided, the neurological legacy of the pandemic continues to demand clinical attention. The symptoms of COVID-19, caused by infection with the SARS-CoV-2 virus, vary widely in both quality and intensity. Noteworthy among these symptoms are the many described neurological manifestations of COVID-19, which can include (though are not limited to) anosmia, ageusia, encephalitis, encephalomyelitis, seizure, Guillain-Barré syndrome, acute cerebrovascular incidents, and critical illness neuropathy [2-5]. More than 30% of SARS-CoV-2 patients present neurological symptoms during COVID-19 [6], with estimates in 2020 suggesting that neurological symptoms were observed in up to 57% of hospitalized patients and showed increasing incidence with the more critically ill cohort [7]. Long COVID is now estimated to affect between 80 and 400 million people globally, with neuropsychiatric symptoms, including cognitive dysfunction, fatigue, and headache, among the most persistent and debilitating manifestations [8-10]. A recent meta-analysis of 125 studies encompassing over 4 million patients confirmed that these neurological symptoms persist well beyond the acute phase and remain prevalent at 12 months or longer after infection [11].

This study was conducted during June-August 2020, a critical period when clinicians were encountering COVID-19 patients during the first major wave in the United States but before formal recognition of long COVID syndrome or establishment of clinical guidelines for long-term neurological monitoring. The absence of such guidance meant that individual clinicians were navigating decisions about neurological follow-up without standardized frameworks, decisions whose downstream effects continue to shape patient outcomes. Capturing clinician attitudes and practices during this early period provides essential baseline data for understanding how clinical care paradigms have since evolved. Although COVID-19 fell out of the top 10 leading causes of death in the United States in 2024 for the first time since 2020 [12], the cumulative population of survivors requiring long-term neurological follow-up continues to grow, making these foundational observations increasingly (not decreasingly) relevant.

SARS-CoV-2 is not unique among human coronaviruses in its demonstrated ability to affect the nervous system. Both SARS-CoV-1 and MERS have been shown to infect the central nervous system [13-15], and similar findings exist for SARS-CoV-2, though whether direct central nervous system invasion plays a common role in COVID-19 neurological manifestations remains an active area of investigation. Proposed routes of CNS invasion include the olfactory nerve, vagus nerve, damage to lung endothelium, and disruption of the blood-brain barrier [9]. The difficulty of establishing a causal link between neurological symptoms and COVID-19 neurotropism is compounded by the confounding effects of severe systemic illness, which can independently cause profound neurological symptoms [16]. Neuropathological evidence to date suggests that COVID-19 neurological symptoms arise from a combination of neurotropic, inflammatory, and endothelial/hypoxic mechanisms [17,18].

Although the acute neurological manifestations of COVID-19 (≤5 weeks from infection onset) have been extensively characterized, the long-term neurological consequences represent a growing and arguably more consequential challenge. The lasting effects of COVID-19 infection became so prevalent that they are collectively referred to as “long COVID” syndrome, describing symptoms persisting ≥12 weeks post-infection, when viral load is undetectable, and symptoms are not explained by alternative diagnoses [10]. The World Health Organization established a clinical case definition for post-COVID-19 condition in October 2021 [19], and in 2024, the National Academies of Sciences, Engineering, and Medicine formally defined long COVID as an infection-associated chronic condition, a systemic disease state with profound consequences that can affect any organ system [20]. The US Centers for Disease Control and Prevention has subsequently published clinical guidance for healthcare providers managing post-COVID-19 conditions [21]. The neurological dimensions of long COVID are particularly concerning: Taquet et al. reported that approximately 33% of COVID-19 patients received a neurological or psychiatric diagnosis within six months of acute infection [22], and the pattern of persistent cognitive dysfunction, frequently described as “brain fog” or “COVID-Brain,” affects over half of long COVID populations surveyed [23,24]. Coronavirus infections also have documented links to the development of multiple sclerosis [25-28], Parkinson’s disease [29], and various neuropsychiatric conditions [30], raising concerns about a delayed wave of neurodegenerative disease among COVID-19 survivors. Long COVID is estimated to affect 31%-69% of individuals with a prior COVID-19 diagnosis [10], and early predictions that the long-term demands for neurological care could far surpass acute pandemic needs [31] have proven prescient as healthcare systems now contend with millions of patients experiencing persistent neurological symptoms.

Despite the now well-documented neurological burden of COVID-19, limited research has examined how clinicians perceived and responded to these risks during the early pandemic, before formal guidelines existed. In this study, we investigated clinician attitudes and practices regarding both acute and long-term neurological care paradigms for COVID-19 patients during June-August 2020. Our survey targeted physicians and advanced practice providers within a single healthcare network in Northwest Ohio, capturing baseline data on the clinical decision-making that preceded, and likely influenced, the subsequent development of long COVID care infrastructure.

This work was previously presented as a poster at the Congress of Neurological Surgeons Annual Meeting on October 11-15, 2025.

## Materials and methods

IRB approval

This study was approved by the University of Toledo Institutional Review Board (IRB) and assigned a protocol number of 300681-UT (exempt status, June 1, 2020). This study met the criteria for exemption as a non-experimental, cross-sectional survey involving no identifiable data collection.

Inclusion and exclusion criteria

This study included physicians and advanced practice providers (APPs; including nurse practitioners, physician assistants, certified registered nurse anesthetists (CRNAs), and similar roles) with current clinical privileges at the University of Toledo Medical Center or ProMedica Health System facilities. The exclusion criteria included non-clinical staff, trainees without independent clinical privileges, and individuals who did not consent to participate after reviewing the informed consent document presented on the first page of the online survey.

Study sample

This survey was distributed to all physicians and APPs indexed in the Medical Staff Services database at the University of Toledo Medical Center and ProMedica Hospitals. This healthcare network includes multiple hospital systems serving diverse urban and rural patient populations across Northwest Ohio and Southeast Michigan, encompassing academic medical centers, community hospitals, and primary care practices. This diversity in practice settings and patient populations within the network provides broader representativeness than a single institution study.

Participant recruitment and study procedures

This was a non-experimental, cross-sectional survey study distributed and hosted online via the Qualtrics survey platform licensed to the University of Toledo. Clinicians were invited to participate anonymously in the survey through three outreach emails sent via the Qualtrics platform (for University of Toledo clinicians) or through the ProMedica Central Verification Office (for ProMedica clinicians) and three announcements published in the University of Toledo College of Medicine and Life Sciences weekly newsletter. These solicitations to participate in the survey were distributed between June 25 and 27, 2020. Survey responses were collected between June 29 and August 27, 2020. This timing captured clinician perspectives during the first major wave of the pandemic in the United States, before widespread recognition of long COVID syndrome and prior to the development of institutional protocols for long-term neurological monitoring. Participation in this study was anonymous; no identifying information was collected, and IP addresses were not recorded.

Survey design and questionnaire development

We used guidance from established survey methodology literature [32], expert consultation, and input from clinical neurology specialists to develop an original 5-10-minute adaptive questionnaire for clinicians (Supplementary material 1). Survey items were reviewed by the research team, which included a neurosurgeon, a cell biologist with expertise in medical education research, and medical students with clinical exposure, and were refined through iterative review for clarity, clinical relevance, and content validity. The questionnaire included 13-26 questions depending on the respondent’s prior experience (i.e., clinicians who had not cared for any COVID-19 patients were not asked about their referral and screening behavior in COVID-19 patients). Participating clinicians were permitted to skip or omit questions. Only completed portions of incomplete submissions were included in the final analysis. For purposes of this study, “long-term neurological follow-up or monitoring” was defined as any clinical monitoring for neurological symptoms or sequelae occurring beyond the acute illness phase, consistent with the timeline later formalized by the WHO (≥12 weeks post-infection) [19]. The survey instrument provided respondents with examples: “neurological specialist care, neuropsychiatric specialist care, primary care with increased vigilance to new onset neurological symptoms, etc.”

Data processing

**Figure 1:**
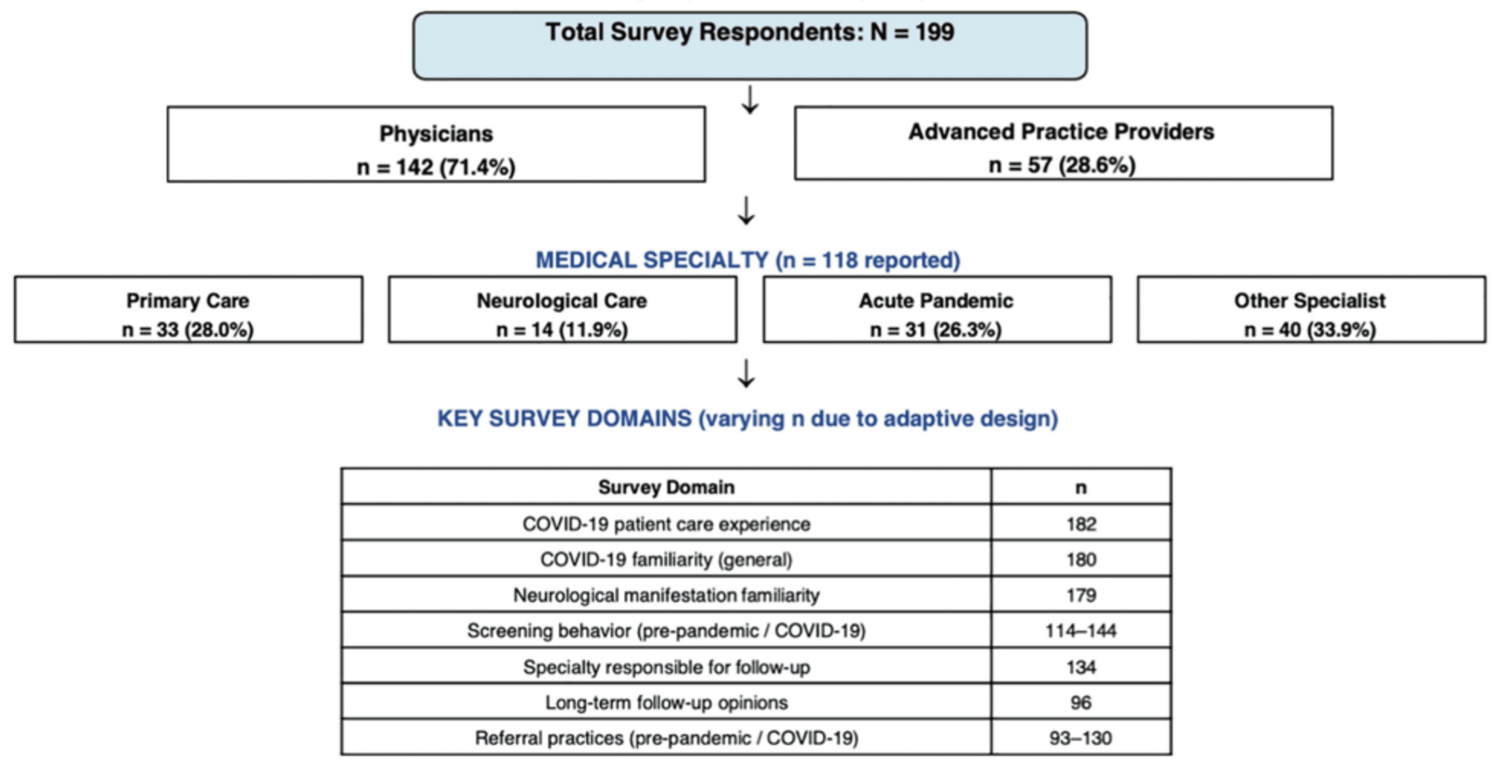
Survey response flow diagram and sample size distribution This flow diagram illustrates the distribution of 199 survey respondents across provider types, medical specialties, and key analytical domains. The survey employed an adaptive design with 13-26 questions per respondent, depending on clinical experience, resulting in varying sample sizes (n = 93-182) across domains. Medical specialties were recoded into four groups: primary care (family medicine, internal medicine, geriatrics, pediatrics); neurological care (neurology, neurosurgery, ophthalmology, psychiatry); acute pandemic care (pulmonary medicine, critical care, emergency medicine, anesthesiology, infectious diseases, hospitalists); and other specialist care (all remaining specialties). Respondents who had not cared for COVID-19 patients were not asked about referral and screening behavior. Only completed portions of incomplete submissions were included in the analyses.

The distribution of respondents across provider types, medical specialties, and analytical domains is shown in Figure 1. Medical specialties were re-coded as follows: primary care specialties include family medicine, internal medicine, geriatrics, and pediatrics; neurological specialties include neurology, neurosurgery, ophthalmology, and psychiatry; acute pandemic specialties include pulmonary medicine, critical care, emergency medicine, anesthesiology, infectious diseases, and hospitalists; other specialties include cardiology, dermatology, endocrinology, gastroenterology, hematology, medical microbiology, medical oncology, nephrology, obstetrics/gynecology, pain medicine, palliative medicine, pathology, pediatric cardiology, pediatric dentistry, pediatric hematology/oncology, physical medicine, radiology, vascular medicine, orthopedic surgery, general surgery, surgical oncology, and urology.

Statistical analysis

Survey variables were recoded using SAS version 9.4 (SAS Inc., Cary, NC, USA) to account for skip patterns and missing responses, as well as grouping write-in responses and text variables into numeric categories. Respondent demographics and study variables were summarized using descriptive statistics. Univariate and bivariate frequencies were generated with SAS Proc Freq. Pearson’s chi-square was used for bivariate frequencies where all table cells had counts greater than five, while Fisher’s exact test was used for bivariate frequencies when one or more cells had counts less than five. p-values ≤ 0.05 were considered statistically significant.

Study outcomes included clinician screening behavior for neurological symptoms, referral rates for long-term neurological follow-up, and clinician attitudes regarding recommended follow-up. Outcomes were summarized descriptively, with bivariate tests (Pearson’s chi-square, Fisher’s exact test) used to examine associations between respondent medical specialty grouping (primary care, neurological care, acute pandemic specialties, other specialist care) and attitudes toward specialty responsibility for long-term care. Respondent demographics, including provider type (physician vs. APP) and years since training completion, were summarized descriptively.

Missing data were handled as follows: only completed portions of incomplete submissions were included in the analysis, skipped items were excluded from the denominator for that specific analysis, and the adaptive survey design routed respondents past questions not applicable to their experience. No imputation methods were used. Variation in sample sizes across analyses reflects the adaptive survey design and permissible question omission; specific denominators are reported for each analysis.

This study is reported in accordance with the Strengthening the Reporting of Observational Studies in Epidemiology (STROBE) guidelines for cross-sectional studies and the Checklist for Reporting Results of Internet E-Surveys (CHERRIES). Completed checklists are provided as in the Appendices (Supplementary materials 2 and 3).

## Results

A total of 199 clinicians participated in this survey. The cohort consisted of 142/199 (71.4%) physicians and 57/199 (28.6%) APPs (Table 1, Figure 1). Respondents were asked to write in their medical specialty, and frequencies of respondents in each medical specialty group were as follows: 33/118 (28.0%) primary care, 14/118 (11.9%) neurological care, 31/118 (26.3%) acute pandemic specialties, and 40/118 (33.9%) other specialist care (Table 1). Most respondents (115/199, 57.8%) had completed their residency or advanced practice training within the past 15 years. First-hand care experience with COVID-19 patients was reported by 150/182 (82.4%) of the respondents (Table 1).

**Table 1:** Respondent demographics and clinical characteristics Demographics, training background, and clinical experience of survey respondents. Medical specialties were recoded into four groups: primary care (family medicine, internal medicine, geriatrics, pediatrics); neurological care (neurology, neurosurgery, ophthalmology, psychiatry); acute pandemic specialties (pulmonary medicine, critical care, emergency medicine, anesthesiology, infectious diseases, hospitalists); and other specialist care (all remaining specialties). Statistical analysis: Pearson’s chi-square test (χ² = 75.43, p < 0.001) was used to compare levels of familiarity with neurological manifestations of COVID-19; Pearson’s chi-square test (χ² = 0.08, p = 0.77) was used to compare proportions of severe vs. non-severe neurological symptom experience. A p-value < 0.05 is considered statistically significant and is marked with an asterisk (*).

Characteristic	Frequency	Percent (%)	Test statistic	p-value
Profession				
Physician	142	71.4	-	-
Advanced practice provider	57	28.6	-	-
Total	199	100	-	-
Medical specialty				
Primary care	33	28.0	-	-
Neurological care	14	11.9	-	-
Acute pandemic care	31	26.3	-	-
Other specialist care	40	33.9	-	-
Total	118	100	-	-
Training completion				
<15 years ago	115	57.8	-	-
15+ years ago	84	42.2	-	-
Total	199	100	-	-
Cared for COVID-19 patients				
Yes	150	82.4	-	-
No	32	17.6	-	-
Total	182	100	-	-
Familiarity with COVID-19 overall				
Extremely familiar	44	24.4	-	-
Very familiar	80	44.4	-	-
Moderately familiar	54	30.0	-	-
Slightly familiar	2	1.1	-	-
Not familiar at all	0	0	-	-
Total	180	100	-	-
Familiarity with neurological manifestations of COVID- 19				
Extremely familiar	14	7.8	χ² = 75.43	<0.001*
Very familiar	35	19.6	-	-
Moderately familiar	65	36.3	-	-
Slightly familiar	52	29.1	-	-
Not familiar at all	13	7.3	-	-
Total	179	100	-	-
Care experience with COVID-19 neurological symptoms				
Yes	107	82.3	-	-
No	23	17.7	-	-
Total	130	100	-	-
Care experience with severe COVID-19 neurological symptoms				
Yes	55	51.4	χ² = 0.08	0.77
No	52	48.6	-	-
Total	107	100	-	-

Nearly all respondents (178/180, 98.9%) self-identified as at least moderately familiar with what was currently known about COVID-19 (Table 1). In contrast, fewer (114/179, 63.7%) respondents self-identified as at least moderately familiar with the neurological manifestations of COVID-19 (Table 1). Among clinicians who cared for COVID-19 patients with neurological symptoms, a majority (55/107, 51.4%) classified the observed neurological complications as severe (Table 1).

When asked about pre-pandemic neurological screening clinical behaviors, 48/114 (42.1%) of respondents indicated that they routinely screened more than 50% of their total patient cohort for neurological symptoms, corresponding to an average reported neurological screening rate of 45.6% (Table 2). This data followed a bimodal distribution, with the most reported screening rate answers being 0% and 100% (Table 2), suggesting that practitioners either consistently incorporated neurological screening into their practice paradigm or did not include it at all. 

**Table 2:** Neurological screening rates: pre-pandemic baseline compared to COVID-19 patients Distribution of clinician-reported neurological screening rates in their total patient population under ordinary (pre-pandemic) circumstances compared to COVID-19 patients at the time of survey (June-August 2020). Statistical test used: Pearson’s chi-square test. A p-value < 0.05 is considered statistically significant.

Screening rate category	Pre-pandemic, n (%)	COVID-19 patients, n (%)	Chi-square	p-value
0%	28 (24.6%)	21 (18.1%)	7.89	0.096
1-25%	22 (19.3%)	12 (10.3%)	-	-
26-50%	16 (14.0%)	14 (12.1%)	-	-
51-75%	9 (7.9%)	13 (11.2%)	-	-
76-100%	39 (34.2%)	56 (48.3%)	-	-
Total	114 (100%)	116 (100%)	-	-

By comparison, the reported neurological screening rate for COVID-19 patients was elevated: responding clinicians indicated that they were screening an average of 59.7% of their COVID-19 patients for neurological symptoms, with 69/116 (59.5%) indicating screening in more than 50% of their COVID-19 patients (Table 3). Most providers (102/144, 70.8%) reported that their screening behavior had not changed in the prior three months, while fewer than a third (42/144, 29.2%) reported that it had increased (Table 3). No providers reported a decrease in screening, suggesting appropriate vigilance to the threat of acute neurological symptoms in COVID-19 patients.

**Table 3:** Detailed neurological screening practices in COVID-19 patients Detailed distribution of clinician-reported neurological screening rates comparing pre-pandemic baseline practices to COVID-19-specific screening behavior at the time of survey (June-August 2020). Statistical tests used: chi-square test for comparison of pre-pandemic vs. COVID-19 screening rates; Fisher’s exact test for change in screening behavior. A p-value < 0.05 is considered statistically significant and is marked with an asterisk (*).

Measure	Frequency	Percent (%)	Test statistic
Percent of total patients normally screened for neurological symptoms (pre-pandemic)			
0%	28	24.6	Chi-square = 7.89
1%-25%	22	19.3	
26%-50%	16	14.0	
51%-75%	9	7.9	
76%-100%	39	34.2	
Total	114	100	p = 0.096
Percent of COVID-19 patients currently screening for neurological symptoms			
0%	21	18.1	
1%-25%	12	10.3	
26%-50%	14	12.1	
51%-75%	13	11.2	
76%-100%	56	48.3	
Total	116	100	
Change in neurological screening rate in COVID-19 patients (past 3 months)			
Increased	42	29.2	Fisher’s exact
Decreased	0	0	
No change	102	70.8	
Total	144	100	p < 0.001*

Within our cohort, nearly half (45/96, 46.9%) of respondents indicated that more than 50% of all individuals infected with SARS-CoV-2 should receive long-term neurological follow-up or monitoring (Table 4). 

**Table 4:** Clinician opinions on the appropriate percentage for long-term neurological follow-up Distribution of respondent opinions regarding what percentage of all SARS-CoV-2 infected individuals should receive long-term neurological follow-up or monitoring. Long-term neurological follow-up was defined as any medical follow-up appropriately conscious of potential neurological sequelae development, including (but not limited to) explicit care by a neurologist or mindful monitoring by a primary care provider. This table presents descriptive statistics only; no comparative statistical tests were performed.

Percentage of COVID-19 patients who should receive follow-up	Frequency	Percent (%)
0%	10	10.4
1%-25%	30	31.3
26%-50%	11	11.5
51%-75%	12	12.5
76%-100%	33	34.4
Total	96	100
Summary statistics		
Mean	48.9%	
Median	50%	
Mode	100%	

On average, providers indicated that 48.9% of individuals infected with SARS-CoV-2 should be followed neurologically in some long-term capacity (Table 5). The most frequently selected response was that 100% of infected individuals should receive long-term neurological follow-up or monitoring (Table 4). 

**Table 5:** Practice-belief disconnect in long-term neurological follow-up for COVID-19 patients Comparison of clinician beliefs about appropriate long-term neurological follow-up for COVID-19 patients versus actual referral practices during the early pandemic period (June-August 2020). Survey respondents included 199 physicians and advanced practice providers across a healthcare network in Northwest Ohio and Southeast Michigan. Long-term neurological follow-up was defined as any medical follow-up appropriately conscious of potential neurological sequelae development in individuals recovering from COVID-19. Statistical test used: Pearson’s chi-square test (χ² = 24.03, p < 0.001) comparing beliefs vs. actual practices. A p-value < 0.05 is considered statistically significant and is marked with an asterisk (*).

Measure	n (%)	Chi-square value	p-value
Clinician beliefs about appropriate care			
Respondents believing >50% of COVID-19 patients should receive long-term neurological follow-up	45/96 (46.9%)	-	-
Mean percentage of COVID-19 patients clinicians believe should receive follow-up	48.9%	-	-
Actual referral practices for COVID-19 patients			
Respondents actively referring >50% of their COVID-19 patients for long-term follow-up	13/93 (14.0%)	24.03	<0.001*
Mean referral rate for COVID-19 patients	19.9%	-	-
Respondents reporting no change in referral practices since pandemic onset	100/130 (76.9%)	-	-
Pre-pandemic baseline referral practices			
Mean referral rate for all patients (pre-pandemic)	14.1%	-	-

Respondents most frequently identified neurology as the specialty that should be primarily responsible for providing this care (66/134, 49.3%), with primary care indicated second most (28/134, 20.9%) (Table 6).

**Table 6:** Specialty assignment for long-term neurological follow-up of COVID-19 patients Respondent perspectives on which medical specialty should be primarily responsible for providing long-term neurological follow-up of COVID-19 patients (n = 134 respondents). *Statistical test used: Fisher’s exact test for association between respondent specialty and choice of responsible specialty. A p-value < 0.05 is considered statistically significant.

Medical specialty responsible	n	Percentage (%)	Test statistic
Neurology	66	49.3%	Fisher’s exact
Primary care	28	20.9%	-
Multiple departments (2+)	23	17.2%	-
Depends on symptoms	13	9.7%	-
Don’t know	4	3.0%	-
Total	134	100%	p = 0.35

The limited size of our survey cohort constrained the ability to draw statistically significant conclusions about the opinions of specific medical specialties. For example, we observed no increase in neurological screening within the neurological care and other specialist care cohorts (Table 7). Among neurological care providers, this is justifiable, considering the already high rate of neurological screening behavior within this group. However, it was difficult to draw meaningful conclusions regarding this observation within the other specialist care group, given the diverse scopes of practice represented.

**Table 7:** Cross-tabulation of recommended responsible specialty by respondent medical specialty group Distribution of respondent opinions regarding which medical specialty should be primarily responsible for providing long-term neurological follow-up of COVID-19 patients, stratified by the respondent’s own medical specialty group. Long-term neurological follow-up was defined as any medical follow-up appropriately conscious of the potential development of neurological sequelae in individuals recovering from COVID-19, including (but not limited to) explicit care by a neurologist or mindful monitoring by a primary care provider. Medical specialty groupings: primary care (family medicine, internal medicine, geriatrics, pediatrics); neurological care (neurology, neurosurgery, neuropsychology); acute pandemic specialties (emergency medicine, critical care, pulmonology, infectious disease, anesthesiology); other specialist care (all remaining specialties). “Unknown” includes respondents who did not provide a medical specialty write-in.

Respondent specialty	Recommended responsible specialty, n (%)					Total
	Neurology	Primary care	2+ departments	Depends on symptoms	Don’t know	
Primary care	9 (37.5%)	8 (33.3%)	2 (8.3%)	4 (16.7%)	1 (4.2%)	24
Neurological care	4 (40.0%)	2 (20.0%)	1 (10.0%)	2 (20.0%)	1 (10.0%)	10
Acute pandemic specialties	11 (50.0%)	6 (27.3%)	3 (13.6%)	2 (9.1%)	0 (0%)	22
Other specialist care	11 (40.7%)	4 (14.8%)	9 (33.3%)	2 (7.4%)	1 (3.7%)	27
Unknown	31 (60.8%)	8 (15.7%)	8 (15.7%)	3 (5.9%)	1 (2.0%)	51
Total	66 (49.3%)	28 (20.9%)	23 (17.2%)	13 (9.7%)	4 (3.0%)	134 (100%)

Responses indicated that prior to March 9, 2020 (the first confirmed COVID-19 case in Ohio was reported March 9, 2020), only 9/102 (8.8%) of clinicians reported referring more than 50% of their total patients for long-term neurological follow-up or monitoring (Table 8). On average, 14.1% of respondents referred their total patient cohort for neurological follow-up, and the most frequent response was a 0% referral rate (Table 5). After March 09, 2020, 13/93 (14.0%) of respondents indicated referring more than 50% of their COVID-19 patients for long-term neurological follow-up or monitoring (Table 5). While this corresponded to a modestly increased average referral rate of 19.9%, the most frequent response remained 0% (Table 8), suggesting minimal change in actual referral practices. This is consistent with the high percentage (100/130, 76.9%) of respondents who indicated that their referral rate for long-term neurological follow-up or monitoring had not changed since March 9, 2020 (Table 5). The contrast between clinician beliefs (46.9% believed most patients should receive follow-up) and actual practices (14.0% actively referring) reveals a substantial practice-belief disconnect at the time (Table 5).

**Table 8:** Detailed long-term neurological referral practices Detailed distribution of clinician-reported referral rates for long-term neurological follow-up or monitoring, comparing pre-pandemic baseline practices to COVID-19-specific referral behavior at the time of survey (June-August 2020). Statistical analysis: Pearson’s chi-square test (χ² = 4.12, p = 0.39) was used for comparison of pre-pandemic vs. COVID-19 referral rate distributions; Fisher’s exact test (p < 0.001) was used for analysis of change in referral behavior over time. A p-value < 0.05 is considered statistically significant.

Measure	Frequency	Percent (%)	Test statistic
Percent of total patients normally referred for long-term neurological follow-up (pre-pandemic)			
0%	37	36.3	Chi-square = 4.12
1%-25%	50	49.0	
26%-50%	6	5.9	
51%-75%	3	2.9	
76%-100%	6	5.9	
Total	102	100	p = 0.39
Percent of COVID-19 patients currently referred for long-term neurological follow-up			
0%	39	41.9	
1%-25%	27	29.0	
26%-50%	14	15.1	
51%-75%	4	4.3	
76%-100%	9	9.7	
Total	93	100	
Change in referral rate for long-term neurological follow-up in COVID-19 patients (past 3 months)			
Increased	27	20.8	Fisher’s exact
Decreased	3	2.3	
No change	100	76.9	
Total	130	100	p < 0.001*

## Discussion

In this study, we characterized clinician-reported behavior and attitudes toward neurological care for COVID-19 patients during the early pandemic (June-August 2020), before formal recognition of long COVID or the establishment of clinical guidelines for long-term neurological monitoring. Our analysis revealed three principal findings: (1) clinicians were substantially more aware of acute neurological manifestations of COVID-19 than of the potential for long-term neurological sequelae; (2) clinician referral rates for long-term neurological follow-up were markedly inconsistent with their stated beliefs regarding the proportion of COVID-19 patients who should receive such care; and (3) there was no consensus regarding which patients should receive long-term neurological follow-up or which medical specialty should be primarily responsible for providing it.

Neurological symptoms are a prevalent and well-established feature of COVID-19, with available evidence from the early pandemic focusing largely on acute neurological symptomology [2,16]. However, the subsequent emergence of long COVID, and its prominent neurological dimensions, has underscored the importance of early baseline data on clinician preparedness for long-term neurological care [9,10,25,26,28,30].

Evolution of clinical practice and persistent infrastructure challenges

Clinical practice has undergone substantial evolution since 2020, including the expansion of telemedicine, establishment of dedicated long COVID clinics, and development of new treatment protocols. However, the fundamental infrastructure challenges identified in our study likely persist in different forms. The core issues we documented-inadequate specialty referral capacity, lack of standardized protocols, and disconnect between clinician awareness and actual practice implementation-represent systemic healthcare delivery problems that transcend specific clinical guidelines. Understanding these foundational challenges provides essential context for current efforts to improve long COVID care delivery systems. Recent evidence confirms that clinician awareness of long COVID remains limited even five years after the pandemic onset, with persistent gaps in knowledge continuing to produce delayed diagnoses and inappropriate treatments that fail to address patients' needs [33].

In 2020, the absence of formal guidance (which began to emerge in 2021 with the WHO clinical case definition [19]) meant that practitioners faced a lack of clarity on what constitutes appropriate long-term neurological assessment of COVID-19 patients. This functionally resulted in the observed scenario in which participating clinicians indicated that approximately 49% of all SARS-CoV-2-infected individuals should receive some form of long-term neurological monitoring but only referred approximately 20% of their COVID-19 patients for such care (Table 5). While nearly half (45/96, 46.9%) of respondents believed more than 50% of COVID-19 patients should receive long-term follow-up, only 13/93 (14.0%) were actively referring at comparable rates (Table 5). This disconnect is especially stark considering that the most frequently selected response was that 100% of COVID-19 patients should receive long-term neurological follow-up (Table 4), strongly suggesting that clinician opinion was not aligned with available clinical practice infrastructure.

The lack of consensus regarding which specialty should be responsible for long-term neurological care presented an additional logistical challenge. Clinicians within our survey cohort most frequently indicated that neurology should be responsible for this care (Table 6). However, with approximately 34 million reported COVID-19 cases in the United States at the time of this 2020 survey, the capacity of approximately 16,000-18,000 practicing neurologists to manage this volume of patients was limited [34]. This concern has been substantiated by a 2024 European Academy of Neurology task force review, which confirmed that the global neurology workforce remains insufficient to meet growing demand, with decreasing numbers of general neurologists and high burnout rates further compounding capacity constraints [35]. Notably, in our study, psychology was rarely suggested as a responsible specialty, despite the substantial psychiatric component of neurological symptoms emerging in long COVID populations [22].

Limitations

Cross-sectional studies such as ours are inherently limited by their inability to evaluate study variables over time. However, the historical value of capturing clinician perspectives during this early pandemic period provides unique insights into baseline clinical thinking before the establishment of formal long COVID care pathways. These early attitudes likely influenced subsequent care patterns and helped explain current variations in long-term neurological follow-up practices. It is compelling to consider how clinician perspectives may have changed in the intervening years since survey administration, particularly given the emergence of variants with increased transmissibility and the continually growing number of individuals surviving acute COVID-19 illness. Efforts are underway at our institution to address this question with a similarly designed follow-up study.

While our findings are drawn from a single healthcare network, the fundamental challenges identified-lack of formal guidelines, limited specialty referral capacity, and resource constraints-likely represent systemic healthcare delivery problems rather than region-specific anomalies. The core issues we documented in 2020 parallel challenges that continue to be reported in long COVID care delivery, supporting the broader relevance of our early findings.

The survey instrument was developed through expert consultation and iterative review but did not undergo formal psychometric validation, which may limit the reliability of individual items. The delay between data collection (2020) and publication reflects multi-institutional data processing, faculty and student transitions across institutions, and the unprecedented disruptions of the pandemic period, rather than concerns about data quality or relevance.

A precise non-response rate cannot be calculated because the total number of clinicians who received recruitment emails through the ProMedica Central Verification Office is not precisely known, as the email contact list or its details could not be shared directly with the research team per institutional policy. The University of Toledo Medical Center Medical Staff Services database contained approximately 800 indexed clinicians; combined with the ProMedica network distribution, the total potential reach is estimated at several thousand clinicians.

The relatively small proportion of neurological specialists in our cohort (14/118, 11.9%) reflects the actual composition of the clinical workforce at the surveyed institutions and limits subgroup analysis for this specialty. However, the study was intentionally designed to survey all clinicians, as long-term neurological follow-up decisions are made across specialties, not solely by neurologists. The perspectives of non-neurological specialists are particularly valuable given our finding that neurology was most frequently identified as the responsible specialty for follow-up care, highlighting the need for cross-specialty coordination.

Notwithstanding these acknowledged limitations, this study illuminates challenges facing clinicians in addressing the long-term neurological burden associated with the COVID-19 pandemic. Until formal guidelines became available [19], clinician opinions remained diverse regarding these issues. The cumulative population of COVID-19 survivors requiring long-term neurological monitoring continues to grow, reinforcing the continued relevance of these foundational findings for healthcare planning and pandemic preparedness. The continuance, evolution, and scope of COVID-19 will require clinicians and public health officials to more broadly consider the long-term neurological complications associated with SARS-CoV-2 infection. With an estimated cumulative global incidence of 400 million individuals affected and an annual economic impact of approximately $1 trillion, a recent comprehensive review has characterized long COVID as a condition demanding sustained investment in research, healthcare infrastructure, and coordinated policy responses [36], reinforcing the urgency of the systemic challenges this study first documented at the clinician level.

## Conclusions

This study provides foundational baseline data on clinician perspectives regarding long-term neurological care for COVID-19 patients, captured during the early pandemic period (June-August 2020) before the establishment of formal clinical guidelines or widespread recognition of long COVID syndrome. Our findings reveal fundamental gaps between clinician awareness and clinical practice implementation, particularly the notable disconnect between clinician beliefs regarding the need for long-term neurological monitoring and actual referral practices. While our data were collected within a single healthcare network in Northwest Ohio, the systemic challenges we identified (lack of standardized protocols, unclear specialty responsibility for long-term care, and insufficient referral infrastructure) likely represent broader healthcare delivery problems rather than region-specific anomalies. These foundational insights remain relevant as healthcare systems continue to manage the ongoing burden of COVID-19 survivors who may require long-term neurological follow-up. Understanding the baseline attitudes and practice patterns documented here provides essential context for evaluating the evolution of long COVID care paradigms and for informing public health strategies to address the long-term neurological burden associated with COVID-19 infection.

## Appendices

Supplementary material 1

Survey questions

Survey completion time: approximately 5-10 minutes.

These survey questions will be administered online through the Qualtrics survey platform licensed to the University of Toledo. Please follow the link below to view this survey online.

Questions:

Part 1 - clinician demographics

1. What is your medical specialty?

_____________________________________________

2. What is your profession?

i. Physician

  When did you finish your residency or fellowship training?

a. I am currently a resident or fellow

b. 0-5 years ago

c. 5-15 years ago

d. 15+ years ago

e. Advanced practice provider

  When did you finish your advanced practice training?

a. 0-5 years ago

b. 5-15 years ago

c. 15+ years ago

Part 2 - clinician viewpoints, general COVID-19

COVID-19 is an illness that was first described on December 31, 2019. COVID-19 is caused by infection with the novel coronavirus (SARS-CoV-2). On March 11, 2020, the World Health Organization officially classified COVID-19 as a global pandemic. As of May 6, 2020, approximately 1.2 million cases of COVID-19 have been reported in the United States.

3. To what extent has the COVID-19 pandemic impacted your everyday medical practice?

a. A great deal

b. A moderate amount

c. A little

d. Not at all

  Survey will adapt to skip questions #4 & 5

4. In what ways has the COVID-19 pandemic impacted your everyday medical practice?

Please check all that apply

a. Increased patient numbers

b. Decreased patient numbers

c. Increased patient complexity

d. Decreased patient complexity

e. Increased reimbursement

f. Decreased reimbursement

g. Increased earning potential

h. Decreased earning potential

i. Increased expenses

j. Decreased expenses

k. Increased demand for technological fluency/telemedicine

l. Decreased demand for technological fluency/telemedicine

m. Increased oversight

n. Decreased oversight

o. Increased managerial responsibilities

p. Decreased managerial responsibilities

q. Increased work hours

r. Decreased work hours

s. Increased job satisfaction

t. Decreased job satisfaction

u. Other:    _______________________

5. Please tell us your additional thoughts about COVID-19’s impact on your medical practice:

___________________________________________________________________________

6. How would you describe the COVID-19-related resources available to you as a clinician?

a. Better than adequate

b. Adequate

c. Neither adequate nor inadequate

d. Inadequate

e. Worse than inadequate

7. Within the context of the COVID-19 pandemic, what additional resources do you feel would improve your ability to do your job?

___________________________________________________________________________

Part 3 - clinician experiences, general COVID-19

Much remains unknown about the possible symptoms, appropriate management, and standard clinical course of COVID-19. Symptoms of COVID-19 can range from mild to severe illness and may include fever, chills, repeated shaking with chills, muscle pain, headache, sore throat, and new loss of taste or smell. This is not an exhaustive list.

For the purposes of this survey, COVID-19 patients include:

i. Patients with a positive COVID-19 laboratory test 

AND/OR

ii. Symptomatic patients with a known exposure to COVID-19 without a more likely alternative diagnosis (CDC expanded case definition of probable COVID-19 diagnoses)

8. How familiar are you with what is currently known about COVID-19?

a. Extremely familiar

b. Very familiar

c. Moderately familiar

d. Slightly familiar

e. Not familiar at all

9. How many COVID-19 patients have you cared for?

  Examples of care may include screening, testing, triage, telemedicine, outpatient care, inpatient care, etc. 

a. 0

  Survey will adapt to skip questions #10 & 12-19

b. 1-5

c. 5-25

d. 25-50

e. 50+ 

10.  What percentage of your patients were:

a. Mildly ill (did not require hospital admission)

b. Moderately ill (required hospital admission without ICU care)

c. Severely ill (required hospital admission with ICU care)

Part 4 - clinician experiences, neuro-COVID-19

Some COVID-19 patients may demonstrate neurological symptoms, such as an altered sense of smell, headache, and dizziness. However, much remains unknown about the possible neurological manifestations of COVID-19, and the clinical understanding of these features is continually evolving.

On March 9, 2020, Ohio announced its first confirmed case of COVID-19.

11.  How familiar are you with what is currently known about the neurological manifestations of COVID-19?

a. Extremely familiar

b. Very familiar

c. Moderately familiar

d. Slightly familiar

e. Not familiar at all

12.  Prior to March 9, what percentage of your total patients did you normally screen for neurological symptoms?

a. __________

b. I don’t know

13.  Currently, what percentage of your COVID-19 patients are you screening for neurological symptoms?

a. __________

b. I don’t know

14.  Since March 9, has the percentage of your COVID-19 patients that you screen for neurological symptoms changed?

a. Yes, it has increased

b. Yes, it has decreased

c. No, it has not changed

15. What percentage of your COVID-19 patients have demonstrated neurological symptoms? 

 a. __________

  If 0%, survey will adapt to skip question #16

b. I don’t know

16.  Collectively, what neurological symptoms have any of your COVID-19 patients demonstrated? 

Please check all that apply

a. Altered sense of smell

b. Ataxia

c. Altered sense of taste

d. Altered consciousness and/or cognition

e. Changes in vision

d. Seizure

e. Headache

f. Acute cerebrovascular incident

g. Dizziness

h. Other:    _______________________

g. Neuralgia

17.  Prior to March 9, for what percentage of your total patients did you normally recommend long-term neurological follow-up or monitoring?

Examples of neurological follow-up or monitoring may include neurological specialist care, neuropsychiatric specialist care, and primary care with increased vigilance to new-onset neurological symptoms.

a. __________

b. I don’t know

18. Currently, for what percentage of your COVID-19 patients are you recommending long-term neurological follow-up or monitoring?

Examples of neurological follow-up or monitoring may include neurological specialist care, neuropsychiatric specialist care, and primary care with increased vigilance to new-onset neurological symptoms.

a. __________

b. I don’t know

19.  Since March 9, has the percentage of your COVID-19 patients that you recommend for long-term neurological follow-up or monitoring changed?

Examples of neurological follow-up or monitoring may include neurological specialist care, neuropsychiatric specialist care, and primary care with increased vigilance to new-onset neurological symptoms.

a. __________

b. I don’t know

Part 5 - clinician viewpoints, neuro-COVID-19

Many individuals who contract the SARS-CoV-2 virus may not display symptoms or access healthcare for this infection.

20. What percentage of all individuals who contract SARS-CoV-2 do you think should receive long-term neurological follow-up or monitoring?

Examples of neurological follow-up or monitoring may include neurological specialist care, neuropsychiatric specialist care, and primary care with increased vigilance to new-onset neurological symptoms.

a. __________

b. I don’t know

21. What medical specialty should be responsible for the long-term neurological follow-up or monitoring of COVID-19 patients?

_____________________________________________

22.  Please share your additional thoughts about the neurological manifestations of COVID-19:

___________________________________________________________________________

Part 6 - conclusions

We are interested in how the COVID-19 pandemic is affecting your life as a clinician.

23.  Please share any additional reflections on your COVID-19 experiences:

___________________________________________________________________________

Supplementary material 2

**Table 9:** STROBE checklist: cross-sectional studies Reference: von Elm E, Altman DG, Egger M, Pocock SJ, Gøtzsche PC, Vandenbroucke JP; STROBE Initiative. The Strengthening the Reporting of Observational Studies in Epidemiology (STROBE) statement: guidelines for reporting observational studies. J Clin Epidemiol. 2008;61(4):344-349.

Item	No.	Recommendation	Reported on page/section
Title and abstract	1	(a) Indicate study design in title or abstract, (b) informative abstract with key results	Title: "Baseline Clinician Attitudes and Practices Regarding Long-Term Neurological Follow-Up for COVID-19 Patients: Early Pandemic Insights With Contemporary Relevance". Abstract: Structured with *Objectives*, *Methods*, *Results*, *Conclusions*, and *Policy implications* subsections; key quantitative findings reported in "n, (%)" format throughout
Introduction			
Background/rationale	2	Explain scientific background and rationale	Introduction paragraphs 1-5: COVID-19 neurological manifestations (including >30% prevalence [6], up to 57% of hospitalized patients [7]); neurotropic potential of SARS-CoV-2; long COVID epidemiology (80-400 million affected globally [8-10]); meta-analysis confirming persistence at 12+ months [11]; NASEM formal definition [20]; literature gap in clinician attitudes
Objectives	3	State specific objectives	Introduction paragraph 6: "We investigated clinician attitudes and practices regarding both acute and long-term neurological care paradigm." Abstract Objectives subsection
Methods			
Study design	4	Present key elements of study design	Methods, Participant Recruitment: " Non-experimental, cross-sectional survey study." Methods, IRB approval
Setting	5	Describe setting, locations, relevant dates	Methods: University of Toledo Medical Center and ProMedica Hospitals, Northwest Ohio and Southeast Michigan; distribution June 25-27, 2020; responses collected June 29-August 27, 2020
Participants	6	(a) Give eligibility criteria, sources, methods of selection	Methods, Inclusion and exclusion criteria: physicians and APPs (including nurse practitioners, physician assistants, CRNAs) with current clinical privileges; exclusion of non-clinical staff, trainees without independent privileges, and non-consenters. Methods, Study Sample: census of medical staff services databases
Variables	7	Define all outcomes, exposures, predictors, confounders	Methods, Statistical Analysis: Study outcomes defined as clinician screening behavior, referral rates, and attitudes regarding follow-up. Primary independent variable: Medical specialty grouping (primary care, neurological care, acute pandemic specialties, other specialist care). Provider type and years since training as demographic variables
Data sources/measurement	8	Describe sources and methods of assessment for variables	Methods, Survey Design: Original adaptive questionnaire via Qualtrics; self-reported screening and referral rates; 5-10 minute estimated completion time; developed using established survey methodology literature [32]. Survey instrument provided in the Appendices
Bias	9	Describe efforts to address potential sources of bias	Discussion, Limitations: Acknowledged self-report bias, recall bias, non-response bias, geographic limitation to single healthcare network, lack of formal psychometric validation, small specialty subgroups, no multivariate analysis, publication delay
Study size	10	Explain how study size was arrived at	Methods, Study Sample: Census of all clinicians indexed in medical staff services databases at two healthcare systems. 199 respondents. UT database contained ~800 indexed clinicians; combined reach estimated at several thousand
Quantitative variables	11	Explain how quantitative variables were handled	Methods, Data Processing: Medical specialties recoded into four groups (primary care, neurological care, acute pandemic specialties, other specialist care); screening and referral rates categorized (0%, 1%-25%, 26%-50%, 51%-75%, 76%-100%). Methods, Statistical Analysis: SAS 9.4 recoding of skip patterns and write-in responses
Statistical methods	12	(a) Describe statistical methods, (b) subgroup analyses, (c) missing data, (d) lost to follow-up, (e) sensitivity analyses	(a) Methods, Statistical Analysis: Pearson’s chi-square (cells > 5) and Fisher’s exact test (cells ≤ 5); p ≤ 0.05 significant; SAS 9.4. (b) Stratification by medical specialty group (Table 7). (c) Methods, Missing Data: completed portions only; skipped items excluded from denominator; adaptive routing; no imputation; specific denominators reported. (d) Not applicable (cross-sectional design). (e) Not applicable
Results			
Participants	13	(a) Report numbers at each stage, (b) reasons for non-participation, (c) flow diagram	(a) Results: 199 total respondents (142 physicians, 57 APPs); varying N per analysis section due to adaptive design (n = 72-199). (b) Discussion, Limitations: Non-response rate discussed; ProMedica email list not shareable. (c) Figure 1: Survey response flow diagram and sample size distribution
Descriptive data	14	(a) Characteristics of participants, (b) number with missing data	(a) Table 1: Demographics, medical specialty, training background, COVID-19 experience, familiarity levels, neurological symptom severity. (b) Varying denominators reported throughout (e.g., n = 180, n = 179, n = 118) reflecting adaptive design and missing data (Figure 1)
Outcome data	15	Report numbers of outcome events	Results: All findings reported with "n (%) format". Tables 1-8 with complete denominators. Key outcomes: Screening rates (Tables 2 and 3), beliefs (Table 4), practice-belief disconnect (Table 5), specialty assignment (Tables 6 and 7), referral practices (Table 8)
Main results	16	(a) Give unadjusted estimates, (b) report category boundaries, (c) consider translating estimates	(a) All tables include test statistics (χ² values) and p-values. (b) Screening/referral rates categorized: 0%, 1%-25%, 26%-50%, 51%-75%, 76%-100%. (c) Summary statistics (mean, median, mode) provided in Tables 2, 4, 5, and 8
Other analyses	17	Report other analyses performed	Cross-tabulations by respondent specialty (Table 7); screening change analysis (Table 3, Section C); referral change analysis (Table 8, Section C); practice-belief disconnect comparison (Table 5, χ² = 24.03, p < 0.001)
Discussion			
Key results	18	Summarize key results with reference to objectives	Discussion paragraph 1: Three principal findings directly linked to study objectives: (1) awareness gap between acute and long-term neurological knowledge, (2) practice-belief disconnect in referral rates, (3) no consensus on patient selection or responsible specialty
Limitations	19	Discuss limitations	Discussion, Limitations (6 paragraphs): Cross-sectional design, single healthcare network, no formal psychometric validation, publication delay, non-response rate, small specialty subgroups (14/118 neurological specialists), no multivariate analysis, geographic scope
Interpretation	20	Give cautious overall interpretation	Discussion: Framed as historical baseline data; acknowledged substantial evolution of clinical practice since 2020 (telemedicine, long COVID clinics, treatment protocols); infrastructure challenges persist; recent evidence confirms ongoing clinician awareness gaps [33]
Generalisability	21	Discuss generalisability	Discussion, Limitations paragraph 2: Single healthcare network; systemic challenges identified (lack of guidelines, limited specialty capacity, resource constraints) likely represent broader healthcare delivery problems rather than region-specific anomalies
Other information			
Funding	22	Give source of funding	Acknowledgements: Internal departmental funds from the Departments of Medical Education and Cell and Cancer Biology at the University of Toledo College of Medicine and Life Sciences

**Table 10:** Checklist for Reporting Results of Internet E-Surveys (CHERRIES) Reference: Eysenbach G. Improving the quality of web surveys: the Checklist for Reporting Results of Internet E-Surveys (CHERRIES). J Med Internet Res. 2004;6(3):e34.

CHERRIES item	Explanation	Our study
Design	Describe survey design	Cross-sectional survey study using an original adaptive questionnaire (Methods, Participant recruitment and study procedures)
IRB approval	IRB approval and informed consent process	University of Toledo IRB Protocol #300681-UT, exempt status (June 1, 2020). Non-experimental, cross-sectional survey involving no identifiable data collection (Methods, IRB approval)
Informed consent	Describe consent process	Consent document presented on first page of Qualtrics survey; continuing to the next page indicated consent. Anonymous participation; no identifiable data collected; IP addresses not recorded (Methods, Participant recruitment and study procedures)
Sampling strategy	Describe sampling strategy	Census survey of all physicians and APPs (including nurse practitioners, physician assistants, CRNAs, and similar roles) indexed in medical staff services databases at University of Toledo Medical Center and ProMedica Hospitals (Methods, Inclusion and exclusion criteria; Study sample)
Contact mode	Describe how participants were contacted	Three recruitment emails via Qualtrics (UT clinicians) or ProMedica Central Verification Office (ProMedica clinicians); three announcements in UTCOMLS Dean’s Update weekly newsletter. Distribution: June 25-July 27, 2020 (Methods, Participant recruitment and study procedures)
Advertising the survey	How was survey advertised?	Email invitations and UTCOMLS Dean’s Update newsletter postings distributed between June 25-July 27, 2020. Survey responses collected June 29-August 27, 2020 (Methods, Participant recruitment and study procedures)
Survey administration	Web/email, platform used	Online via Qualtrics survey platform licensed to the University of Toledo (Methods, Participant recruitment and study procedures)
Type of e-survey	Open vs. closed survey	Closed survey: accessible only via direct email link distributed to eligible clinicians (Methods, Participant recruitment and study procedures)
Adaptive questioning	Use of adaptive questioning	Yes. 13-26 questions depending on respondent’s prior experience (e.g., clinicians without COVID-19 patient care experience were not asked about referral and screening behavior in COVID-19 patients) (Methods, Survey design and questionnaire development)
Number of items	Number of survey items	13-26 items (adaptive) organized in sections covering: demographics, COVID-19 experience, neurological familiarity, screening behavior, long-term follow-up opinions, and conclusions (Methods, Survey design and questionnaire development)
Number of screens	Number of web pages	Multiple pages; exact count dependent on adaptive routing (Methods, Survey design and questionnaire development)
Completeness check	Could respondents review/change answers?	Yes. Respondents could navigate back and review/change responses before final submission. Respondents could quit and return to the survey at any time (Methods, Survey design and questionnaire development)
Review step	Was a review step included?	Yes. Qualtrics allows back-navigation for review before submission (Methods, Survey design and questionnaire development)
Unique site visitor	Methods to prevent multiple entries	Anonymous survey; no login required. IP address recording was disabled to ensure anonymity. Multiple submissions from the same individual were possible but unlikely given the professional clinical audience (Methods, Participant recruitment and study procedures)
View rate	Ratio of unique visitors who viewed the survey	Not calculable. ProMedica email list could not be shared with research team per institutional policy; exact number of email recipients unknown (Discussion, Limitations)
Participation rate	Ratio of those who agreed to participate	199 clinicians completed at least a portion of the survey out of the eligible population across two healthcare systems. UT Medical Center Medical Staff Services database contained approximately 800 indexed clinicians; combined with ProMedica network, total potential reach estimated at several thousand (Discussion, Limitations)
Completion rate	Ratio who completed the survey	Variable by section due to adaptive design and permissible skipping. Denominators range from 72 to 199 across different analyses. Only completed portions of incomplete submissions were included. Un-submitted responses collected after 1 month of inactivity (Methods, Missing data; Figure 1)
Cookies used	Were cookies used to identify respondents?	No. Survey was anonymous. No cookies used for identification (Methods, Participant recruitment and study procedures)
IP check	IP address used to identify potential duplicate entries?	No. IP addresses were not recorded to protect respondent anonymity (Methods, Participant recruitment and study procedures)
Log file analysis	Was log file analysis used?	Not applicable. Anonymous survey design (Methods, Participant recruitment and study procedures)
Registration	Was registration required?	No registration required. Survey accessed via direct link in recruitment email (Methods, Participant recruitment and study procedures)
Handling of incomplete questionnaires	How were incomplete questionnaires handled?	Only completed portions of incomplete submissions were included in analysis. Skipped items excluded from denominator for that specific analysis. Un-submitted responses collected after 1 month of inactivity. No imputation methods used. Variation in sample sizes across analyses reflects adaptive design and permissible question omission; specific denominators reported for each analysis (Methods, Missing data)
Questionnaire development	How were items developed?	Developed using guidance from established survey methodology literature (Dillman et al., 2014 [32]), expert consultation, and input from clinical neurology specialists. Items reviewed by research team (neurosurgeon, cell biologist/medical education researcher, medical students with clinical exposure) and refined through iterative review for clarity, clinical relevance, and content validity. No formal psychometric validation (Methods, Survey Design and Questionnaire Development; Discussion, Limitations)
Pre-testing	Was questionnaire pre-tested?	Items reviewed and refined through iterative team review. No formal pilot study in a subpopulation (Methods, Survey Design and Questionnaire Development; Discussion, Limitations)
Statistical correction	Methods for statistical correction	No weighting or correction applied. Bivariate associations tested with Pearson’s Chi-Square (cells >5) or Fisher’s Exact Test (cells ≤5). p≤0.05 considered statistically significant. SAS version 9.4 used for all analyses (Methods, Statistical Analysis)
